# A Shake up

**Published:** 1879-01

**Authors:** 


### "A SHAKE UP."

A fair separation, a thorough sifting has taken place—that's all. Truth may be discovered in time, if we look through either end of the telescope—and whether in dwarfed or monstrous proportions, a lie is certain to be found out, though it stand ever so much in the light of its opposite. It often happens that to get at the truth, clearly and unmistakably defined, the husk containing it must be 'thrashed,' the flail must be used, and the straw and the chaff beaten away from it. It is essential to purification that some things should be 'well shaken,' roughly handled, and exposed to the light of open day.

Stripped of 'borrowed livery,' completely unmasked, and many of the financial marts appeared for the time being to be the devil's camping-ground. Some savings institutions, supported by small depositors whose dimes and whose occasional dollars, were enough to make the banks rich with their earnings—some of these went down in the maelstrom of speculation.

The custodians of sacred trust funds had indulged in 'riotous living' at the expense of the provident and economical.

"And so all through the past year, and even now, the winnowing process, the shaking up and the turning over continues, and the end is not yet. The probing and sifting is thorough, however, and communities, as well as individuals, are demanding full weight and honest measure. Bubbles have been pricked, chaff has been swept away, and there is a cleaner and a better foundation to build upon. Confidence is restored to a man—not when he is sinking, but when he has touched bottom; and it is the credit system, after all, that is the root of the evil. Pay for what you buy, and buy only what you can pay for, and the ills that flesh or business is heir to, will be so materially lessened, that quackery and dishonesty will starve to death for want of their natural nourishment."

